# Quarantining: a mentally distressful but physically comfortable experience in South Korea

**DOI:** 10.1186/s12955-022-02051-4

**Published:** 2022-10-17

**Authors:** Hye-Young Kwon, Yongjoo Kim, Seung-Young Lee, Chang-Bo Kim

**Affiliations:** 1 Division of Biology and Public Health, Mokwon University, Daejeon, South Korea; 2 College of Korean Medicine, Sangji University, Wonju, South Korea; 3 Department of Nursing Science, Kyungbuk College, Seoul, South Korea; 4 Seoul Health Foundation, Seoul, South Korea

**Keywords:** Quarantine, COVID-19, Quality of life, EQ-5D, Republic of Korea

## Abstract

**Objective:**

Quarantine is the first response to the COVID-19 pandemic. Restricting daily life can cause several problems. This study aimed to measure the impact of the COVID-19 quarantine on health-related quality of life (HRQoL) by comparing to the pre-pandemic.

**Methods:**

HRQoL during COVID-19 quarantine was surveyed online using EQ-5D index and matched to that of the pre-pandemic-extracted from nationwide representative data of the Korea Community Health Survey- with propensity scores. A beta regression for the EQ-5D scores and a logistic analysis for individual dimensions of the EQ-5D index were performed to measure the impact of the COVID-19 quarantine on health utility.

**Results:**

The overall scores of the EQ-5D index were significantly higher in the group under quarantine during the COVID-19 pandemic (0.971 SD 0.064) than those before the pandemic (0.964 SD 0.079, Diff. 0.007 SD 0.101, p = 0.043). The beta regression for the overall scores of EQ-5D revealed that quarantining during the COVID-19 pandemic increased by 52.7% compared to normal life before the outbreak(p = 0.045). Specifically, “Depression/Anxiety” deteriorated significantly during quarantining (OR = 0.62, 95% CI:0.48–0.80). However, “Pain/Discomfort” and “Mobility” significantly improved (OR = 5.37, 95% CI:3.71–7.78 and OR = 2.05, 95% CI:1.11–3.80, respectively).

**Conclusion:**

Although the world is facing a challenging moment that it has never been through before, mandatory quarantine has served as an experience that provided mental distress but physical comfort in the Korean context.

## Introduction

With a worldwide death toll of 1,852,392 and more than 85 million infected as of January 4, 2021 [1], the SARS-CoV-2 virus that causes COVID-19 has had a substantial impact on the history of humankind. Since the first outbreak was reported in China in December 2019, the world is still struggling to contain this new infectious disease.

Quarantine is often the first response to be imposed against new infectious diseases [2]. The World Health Organization (WHO) recommended that contacts of patients with laboratory-confirmed COVID-19 be quarantined for 14 days [3]. Health authorities in many countries have since implemented quarantines to limit the spread of the virus. In particular, amid the ongoing COVID-19 pandemic, where a considerable portion of infectious cases are known to be asymptomatic and symptom-based control would not be sufficient [4, 5], the importance of quarantine in a timely manner is stressed [6]. Approximately three million people have been isolated under preventive advisories in South Korea [7], and globally, many countries have either enforced self-isolation or imposed lockdown measures.

The goal of quarantining, whether mandatorily or voluntarily, is to isolate individuals who have potentially been exposed to a contagious disease. This has utility for highly transmissible diseases. However, concerns have been raised on quarantines imposed in ways that are too stringent or haphazard [2, 3, 8]. Suicidal attempts among quarantinees have been reported in South Korea [9]. The WHO has released a guide on implementing quarantine measures for individuals that should be part of a comprehensive package of public health response and containment measures; measures should be fully respectful of the dignity, human rights, and fundamental freedoms of citizens [3]. Indeed, this measure disrupting daily living affects many facets of life, including not simply socioeconomic status but also mental and emotional health [10–13]. Several studies have explored the impact of quarantine on health-related quality of life (HRQoL) [14, 15].

HRQoL is a multidimensional concept that includes aspects related to physical, mental, emotional, and social functioning. It extends beyond direct measures of population health, life expectancy, and causes of death, and focuses on the impact of health on quality of life [16–18]. By limiting daily activities during the COVID-19 pandemic, one would expect the HRQoL of the subjects of quarantine to be affected since HRQoL refers to an individual’s satisfaction or happiness with aspects of life [16–18]. However, the impact of quarantining during the COVID-19 pandemic on HRQoL has not been sufficiently explored. Previous studies have focused on HRQoL before and after the COVID-19 outbreak[14], or HRQoL during quarantine without comparison[15]. Therefore, there is a need for a study that investigates HRQoL among people under quarantine during the COVID-19 pandemic and compares it to data on the general population before the pandemic to analyze factors that contribute to HRQoL.

## Methods

### Study population

#### Sample population under quarantine during the COVID-19 pandemic

Since the outbreak of COVID-19, the Korean government imposed a 14-day quarantine together with the 3T strategy (test, trace, treat) for those who were exposed to confirmed cases and in-bound travelers in the early stage [19, 20]. All preventive measures, including quarantine and screening spots, were managed by local district councils and local community public health centers in local governments coordinating with central governments. The Project of Seoul COVID-19 Study for Quarantine (SCS-Q) was jointly conducted by the Seoul Health Foundation with the affilicated districts of Seoul Metropolitan Government. Accordingly, people living in Seoul Metropolitan City, aged 19 years or older, under a two-week quarantine from October to November 2020, were the subjects of this study.

Quarantinees voluntarily participated in the cross-sectional online survey. A total of 5,175 people under quarantine from October to November 2020 were asked to participate in this online survey. Response to the survey was conditional to the subject’s consent. In total, 1,139 (22.0%) agreed to complete the questionnaire.

#### Control population before the COVID-19 pandemic

To measure the impact of quarantine on HRQoL during the COVID-19 pandemic, a comparison group was established from nationally representative data from the Korea Community Health Survey (KCHS), which has been conducted annually since 2008 on a target population of adults aged 19 years or older [21]. Given that the KCSH includes standardized and validated questionnaires to assess community health status, such as EuroQol-5-Dimensions (EQ-5D) for HRQoL, we selected a group of people similar to the sample case from the KCHS 2019 conducted from August to October 2019 before the COVID-19 pandemic. To do so, the propensity score matching (PSM) technique [22, 23] was employed to pair the quarantinees with participants of the KCHS who are most like them in accordance with the propensity scores computed as a function of individual characteristics such as sex, age, dwelling district, income, working status, education, and hypertension as predisposing diseases. These covariates were selected based on their significance by performing logistic regression with a stepwise selection option.

### Health-related quality of life

Generalized HRQoL instruments are designed to be applicable across all diseases or conditions, different medical interventions, and a wide range of populations [24, 25]. In this study, the EQ-5D, a widely used generic instrument of HRQoL, was used to survey HRQoL among quarantinees during the COVID-19 pandemic. The study used the validated Korean version of the questionnaire [26, 27]. The EQ-5D, with a range of 0 to 1 representing death to perfect health, comprises five questions on mobility, self-care, usual activities, pain or discomfort, and psychological status with three possible answers for each item (1 = no problem, 2 = moderate problem, 3 = severe problem). Responses to individual dimensions of the EQ-5D were also explored.

### Statistical analysis

Categorical variables are expressed as frequencies and percentages, and continuous variables are expressed as means and standard deviations (SD). Chi-square test for categorical variables and Student’s t-test for means were performed. The Mann-Whitney median test for continuous variables and Fisher’s exact test for categorical variables were performed where appropriate.

Factors contributing to the overall EQ-5D scores, calculated based on the Korean Tariff [26, 27], were analyzed with a beta logit distribution considering ceiling effects and anticipated violations of normality and homoscedasticity [28, 29]. Since the EQ-5D scores range from 0 to 1, the bounded variables were rescaled for beta regression [30, 31]. In addition, each domain of the EQ-5D was dichotomized into “no problem” versus “any problems” and analyzed with logistic regression adjusting for socioeconomic and health-related variables. All statistical analyses were performed with SAS 9.4 software (SAS Institute, Inc., Cary, NC).

### Ethical statement

This study was approved by the institutional review board of the Seoul Metropolitan City (IRB No. 2020-10-0001). All participants provided informed consent online before initiating the survey.

## Results

### Basic characteristics

The characteristics of the two groups, those under quarantine during the pandemic versus the pre-pandemic group before and after matching based on propensity scores are compared in Table 1. A total of 1,139 quarantinees during the COVID-19 pandemic were compared to 3,649 persons from the KCHS before the COVID-19 pandemic, and revealed statistically significant differences for each covariate. Significant covariates were selected and propensity scores were computed using stepwise logistic regression. After matching, 919 for each group were finally selected; there was no difference in the matched variables between the two groups.

**Table 1:** Study populations based on propensity score matching: pre- and post-matching HRQoL between the two groups

Variables	Before matching						After matching				
	Quarantinees during COVID-19 pandemic (N = 1139)		Control group (N = 3649)		P-value		Quarantinees during COVID-19 pandemic (N = 919)		Control group (N = 919)		P-value
Male, N (%)	565	(49.6%)	1,528	(41.9%)	< 0.0001		468	(50.9%)	468	(50.9%)	NS
Age, Mean (SD)	39.01	(12.54)	52.35	(17.60)	< 0.0001		39.82	(12.16)	40.10	(13.14)	NS
Age group											NS
19 to 40	608	(53.4%)	941	(25.8%)	< 0.0001		472	(51.4%)	466	(50.7%)	
40 to 65	508	(44.6%)	1,690	(46.3%)			427	(46.5%)	433	(47.1%)	
65 and over	23	(2.0%)	1,018	(27.9%)			20	(2.2%)	20	(2.2%)	
Dwelling district					< 0.0001						NS
Nowon	330	(29.0%)	912	(25.0%)			256	(27.9%)	258	(28.1%)	
Seongbuk	341	(29.9%)	917	(25.1%)			276	(30.0%)	276	(30.0%)	
Eunpyeong	261	(22.9%)	910	(24.9%)			208	(22.6%)	204	(22.2%)	
Yangcheon	207	(18.2%)	910	(24.9%)			179	(19.5%)	181	(19.7%)	
Income					< 0.0001						NS
Lowest	138	(12.1%)	146	(4.0%)			26	(2.8%)	29	(3.2%)	
Employment status					< 0.0001						NS
Salaried worker	625	(54.9%)	1,620	(44.4%)			576	(62.7%)	554	(60.3%)	
Self-employed/Employer	98	(8.6%)	407	(11.2%)			86	(9.4%)	115	(12.5%)	
Unemployed	312	(27.4%)	1,588	(43.5%)			241	(26.2%)	238	(25.9%)	
Others	104	(9.1%)	34	(0.9%)			13	(1.4%)	12	(1.3%)	
Education					< 0.0001						NS
High school or less	254	(22.3%)	1,850	(50.7%)			155	(16.9%)	151	(16.4%)	
Tertiary education	885	(77.7%)	1,799	(49.3%)			764	(83.1%)	768	(83.6%)	
Predisposing disease											NS
Hypertension	96	(8.4%)	1,017	(27.9%)	< 0.0001		83	(9.0%)	88	(9.6%)	
Propensity scores, Mean (SD)	0.38	(0.21)	0.19	(0.15)	< 0.0001		0.32	(0.13)	0.32	(0.13)	NS

Table 2 shows that the overall scores of the EQ-5D index were significantly higher in the group under quarantine during the COVID-19 pandemic (0.971 SD 0.064) than those before the pandemic (0.964 SD 0.079, Diff. 0.007 SD 0.101, p = 0.043). In addition, it shows differences in EQ-5D index scores between the two groups tested by socioeconomic characteristics or health states such as predisposing chronic diseases and self-ranked health conditions. As a result, it was found that the socioeconomically vulnerable groups such as those aged 40 or older (Diff. 0.017 SD 0.083, p = 0.002), those in the lowest income class (Diff. 0.112 SD 0.192, p = 0.030), economically inactive (Diff. 0.025 SD 0.098, p = 0.006), less educated (Diff. 0.045 SD 0.123, p = 0.001), and the divorced or widowed (Diff. 0.061 SD 0.109, p = 0.010) showed a significant increase in EQ-5D scores under quarantine during pandemic than pre-pandemic. The scores of those with predisposing diseases such as hypertension (Diff. 0.025 SD 0.080, p = 0.038) and diabetes (Diff. 0.051 SD 0.101, p = 0.035), or who rated their health as “not good” (Diff. 0.019 SD 0.081, p = 0.0002) were significantly higher in the quarantining group than in the pre-pandemic group. In contrast, among those who evaluated their health status positively, their EQ-5D scores were significantly lower in quarantine during pandemic than in the control group (Diff. -0.008 SD 0.056, p = 0.030).

**Table 2:** **Difference in EQ-5D index scores between quarantinees during pandemic versus control group, mean (standard deviation)**

Parameters	Quarantinees during pandemic (N = 919)		No quarantine before pandemic (N = 919)		Differences*		P-value
Overall EQ-5D scores	0.971	(0.064)	0.964	(0.079)	0.007	(0.072)	0.045
By sex							
Male	0.978	(0.070)	0.972	(0.080)	0.006	(0.074)	NS
Female	0.964	(0.060)	0.957	(0.080)	0.008	(0.070)	NS
By age							
19 to 40	0.968	(0.063)	0.973	(0.059)	-0.005	(0.061)	NS
40 to 65	0.972	(0.067)	0.955	(0.096)	0.017	(0.083)	0.002
65 and over	1.000	(0.000)	0.955	(0.070)	0.045	(0.049)	0.010
Dwelling District							
Nowon	0.964	(0.076)	0.970	(0.099)	-0.005	(0.089)	0.001
Seongbuk	0.975	(0.061)	0.962	(0.062)	0.014	(0.062)	0.000
Eunpyeong	0.970	(0.055)	0.965	(0.067)	0.005	(0.061)	NS
Yangcheon	0.975	(0.967)	0.961	(0.950)	0.015	(0.065)	NS
By Income level							
Lowest	0.946	(0.077)	0.833	(0.254)	0.112	(0.192)	0.030
Middle or High	0.972	(0.064)	0.968	(0.062)	0.003	(0.063)	NS
By employment status							
Salaried worker	0.973	(0.053)	0.974	(0.053)	-0.001	(0.058)	NS
Self-employed/Employer	0.971	(0.064)	0.964	(0.070)	0.007	(0.067)	NS
Economically inactive	0.965	(0.070)	0.941	(0.120)	0.025	(0.098)	0.006
Others	0.976	(0.047)	0.965	(0.074)	0.011	(0.061)	NS
By education							
High School or Less	0.961	(0.058)	0.916	(0.149)	0.045	(0.123)	0.001
Tertiary education	0.973	(0.089)	0.974	(0.051)	-0.001	(0.055)	NS
By Marital status							
Married	0.973	(0.067)	0.967	(0.063)	0.005	(0.065)	NS
Single	0.968	(0.061)	0.969	(0.079)	-0.001	(0.071)	NS
Divorced/Widowed	0.969	(0.052)	0.908	(0.133)	0.061	(0.109)	0.010
By self-rated health							
Good/Very good	0.975	(0.068)	0.984	(0.040)	-0.008	(0.056)	0.030
Moderate/Bad/Very bad	0.967	(0.060)	0.948	(0.098)	0.019	(0.081)	0.000
By Hypertension							
Yes	0.974	(0.058)	0.949	(0.097)	0.025	(0.080)	0.038
No	0.971	(0.065)	0.966	(0.077)	0.005	(0.071)	NS
By Diabetes							
Yes	0.980	(0.054)	0.928	(0.137)	0.051	(0.101)	0.035
No	0.971	(0.065)	0.966	(0.076)	0.005	(0.070)	NS

Footnote: Differences were calculated by subtracting EQ-5D scores of the control group from those of cases

### Five dimensions of EQ-5D

**Fig. 1:**
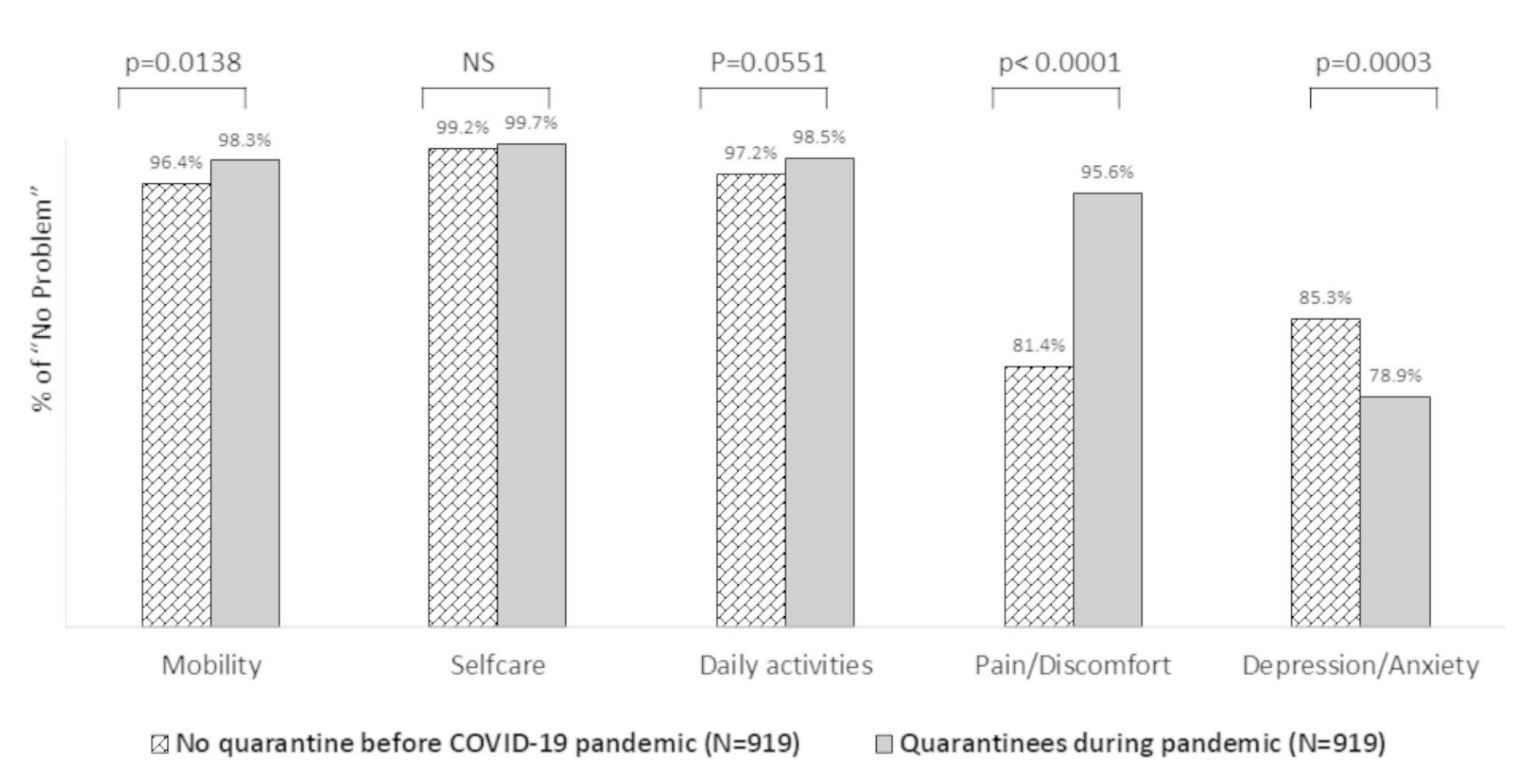
Comparison of EQ-5D’s five dimensions (“no problem” vs. “any problems”) between the two comparison groups

As shown in Fig. 1 after decomposing and analyzing the five domains of the EQ-5D index after dichotomizing into “no problem” or “any problems,” an increasing tendency of “no problem” was found in the quarantined group in all dimensions except psychological status, although it was not significant in “Self-care” and marginally significant in “Usual activities” (p = 0.0551). In particular, the proportion of those who answered “no problem” in the domain “Pain/Discomfort” was higher in the quarantined group than in the comparison group (95.6% vs. 81.4%, p < 0.0001). On the other hand, there was a significant decrease in reporting “no problem” for “Depression/Anxiety” in the quarantined group vs. control group, as expected, (78.9% vs. 85.3%, p = 0.0003) (Fig. 1). In fact, the higher proportion of those reporting “no problem” in the quarantined group resulted in a significant increase in overall EQ-5D scores compared to those in normal life before the pandemic.

### Factors contributing to EQ-5D

Table 3 presents the results of a multivariate regression with a beta logit distribution on overall scores of the EQ-5D index. The estimate of quarantining during the COVID-19 pandemic increased by 52.7% (exp(0.423) = 1.527, p = 0.045) compared to normal life before the outbreak. Women (-12.3% vs. men, p = 0.007), the lowest income class (-29.6% vs. the lowest, p = 0.012), the less educated (-12.1% vs. tertiary education, p = 0.047), and unhealthy people (-17.9% vs. healthier, p < 0.0001) were associated with the lowest HRQoL. Married participants’ scores were 32.4% higher than the divorced or widowed (p = 0.045).

**Table 3:** Results of multivariate beta regression for EQ-5D scores

Parameters			Estimate	S.E	Pr > |t|
Intercept			3.258	0.214	< 0.0001
Quarantine during pandemic	Yes		0.423	0.211	0.045
	(Ref = No quarantine)		-	-	-
Sex	Female		-0.131	0.049	0.007
	(Ref = male)		-	-	-
Age	19 to 39		-0.027	0.167	0.871
	40 to 64		-0.088	0.162	0.587
	(Ref = 65 and over)		-	-	-
Dwelling district	Nowon		0.074	0.069	0.285
	Seongbuk		0.037	0.068	0.582
	Eunpyeong		0.033	0.073	0.655
	(Ref = Yangcheon)		-	-	-
Marital status	Married		0.280	0.140	0.045
	Single		0.289	0.151	0.056
	(Ref = Divorced/widowed)		-	-	-
Income	Lowest		-0.351	0.135	0.012
	(Ref = Middle or High)		-	-	-
Education level	High School or less		-0.129	0.065	0.047
	(Ref = Tertiary)		-	-	-
Employment status	Employer/Self-employed		-0.035	0.077	0.653
	Econ. Inactive		-0.108	0.058	0.065
	No answer		-0.008	0.202	0.970
	(Ref = Salaried workers)		-	-	-
Self-rated health state	Moderate/Bad/Very bad		-0.198	0.047	< 0.0001
	(Ref = Very Good/Good)		-	-	-

More specifically, a logistic analysis was performed to identify the impact of quarantine on each dimension of the EQ-5D. As tabulated in Table 4, the domains most affected by quarantine were “Pain/Discomfort,” “Mobility,” and “Depression/Anxiety.” For “Pain/Discomfort,” quarantining itself had the biggest positive impact and was 5.37 times more likely to be “no problem” (95% CI:3.71–7.78) than the control group. Quarantining also showed a significant and positive impact on “Mobility” (OR = 2.05 for “no mobility-related problem”, 95% CI:1.11–0.3.80). In contrast, it had a negative impact on “Psychological status” (OR = 0.62 for “no depression/anxiety-related problem”, 95% CI:0.48–0.80). In addition, covariates such as sex, income, education, and self-ranked health were significantly associated. Females, those in the lowest income class, those economically inactive such as the unemployed, housewives, or students, the less educated, and those who perceived themselves as unhealthy tended to be less likely to report “no problem” for each domain.

**Table 4:** Results of multivariate logistic regression for the five dimensions of EQ-5D (Event=“No problem”)

Variables		Mobility			Self-care				Daily activities					Pain/Discomfort				Depression/Anxiety			
		OR	95% CI		OR	95% CI			OR		95% CI		OR		95% CI				OR	95% CI	
Quarantine	Yes (Ref = No)	2.05	[1.11-	3.80]										5.37		[3.71-	7.78]	0.62		[0.48-	0.80]
Sex	Female (Ref = male)				6.30		[1.24-	32.13]						0.64		[0.46-	0.89]	0.43		[0.33-	0.56]
Age group	19 to 39																	0.12		[0.02-	0.88]
	40 to 64																	0.13		[0.02-	0.99]
	(Ref = 65 and over)																				
Dwelling District																					
(Ref:Yangcheon)	Nowon													2.19		[1.34-	3.56]				
	Seongbuk																				
	Eunpyeoung																				
Income	Lowest				0.10		[0.01-	0.69]										0.40		[0.22-	0.72]
	(Ref = Middle or High)																				
Education level	High School or Less	0.29	[0.15-	0.53]	0.21		[0.05-	0.81]	0.25	[0.13-		0.50]		0.53		[0.37-	0.77]	0.60		[0.44-	0.83]
	(Ref = Tertiary)																				
Employment status	Employer/Self-employed																				
	Economically inactive	0.48	[0.24-	0.96]					0.34	[0.16-		0.73]									
	Others																				
	(Ref = Salaried worker)																				
Self-rated health	Bad/Very bad																				
	(Ref = Good/Very good)	0.44	[0.23-	0.87]					0.35	[0.16-		0.77]		0.35		[0.25-	0.50]	0.54		[0.41-	0.70]

## Discussions

This study identified the impact of quarantine during the COVID-19 pandemic on HRQoL when compared with quality of life before the outbreak, using a propensity score matching technique in the Korean context. There is a dearth of research exploring HRQoL during the pandemic. One study found the COVID-19 pandemic had an insignificant impact on HRQoL among patients with cardiovascular disease when compared with the pre-pandemic period [14]. Ping et al. (2020) explored HRQoL without this comparison but found that the most frequently reported problems were “Pain/Discomfort” (19.0%) and “Anxiety/Depression” (17.6%) in China [15]. However, there was no study that focused on quarantining and HRQoL in a comparison setting before and during the COVID-19 outbreak. Interestingly, our results found a significantly positive impact of quarantining on HRQoL, particularly, the reporting of problems in “Pain/Discomfort” was significantly lower, which contradicts the findings of Ping et al. [15] but complies with those of Lim et al. [14], which were not significant, though. As expected, “Depression/Anxiety” was affected by not only the quarantine itself [10] but also the situation of the COVID-19 pandemic [12]. As we compared people under quarantine during the pandemic with those before the pandemic, the effect of quarantine in this study was entangled with the quarantine itself as well as the COVID-19 pandemic. However, given the results of Lim et al. (2020) that there was no meaningful change in overall EQ-5D scores before and during the COVID-19 pandemic [14], it was interpreted with caution that the HRQoL of South Koreans improved significantly during quarantine, which caused some mental distress but allowed greater physical comfort. A possible explanation for the greater physical comfort for Koreans would be that after having a negative test, a 14-day quarantine period may be considered a break that provides temporary physical rest, especially in a society where diligence is highly valued. Korea is known for its work culture and the demands on labor, which sometimes leads to death by overworking [32]. In fact, South Korea’s total annual working hours (1,967 h) was ranked 3rd after Mexico (2,137 h) and Costa Rica (2,060 h) among OECD countries in 2019 [33]. Another explanation would be that quarantinees become less physically active since they must stay at home. Some laziness or even sedentariness can be enjoyable; in addition, there would be few physically distressing things happening, especially at home. Apart from quarantine, the findings seem to support previous HRQoL studies [34–37]: people with limited socioeconomic resources, such as women, low-income people, and poorly educated people, have a lower HRQoL.

Several limitations of this study should be noted. First, the study population was biased due to the nature of the online survey that easily omits the elderly who are more vulnerable to the COVID-19 pandemic. In addition, quarantinees living in Seoul participated in this survey Thus, the results of this study may not be representative of the entire population or all age groups in South Korea. In particular, individuals residing in other cities with poor housing conditions, may not experience the same level of physical comforts as those included in our analysis.

Second, as described earlier, our findings of differences in depression/anxiety observed before and after the pandemic may be partly, but not solely, due to the quarantine, as the pandemic per se may partly contribute to the difference. Third, the results may change depending on the magnitude of the incidence. At the time of the survey, the incidence of COVID-19 (87.1 to 256.3 new cases per day) was not as high as at the end of December 2020 where a third surge caused more or less 1,000 confirmed new cases per day and the highest incidence rate since the outbreak [38]. In this situation, the effect of the pandemic itself could increase and HRQoL might be negatively affected. In contrast, as quarantine reduces the risk of infection by preventing contact with people, the positive effects of quarantine could be highlighted. Further studies that are more systematic are required. Despite these limitations, to our knowledge, this study is the first to investigate the effect of quarantine during the COVID-19 pandemic compared to the pre-pandemic in South Korea.

## Conclusions

In conclusion, this study confirmed that, contrary to our expectations, quarantining during the pandemic was perceived as causing psychological distress but simultaneously providing greater physical comfort in the Korean context.

## Data Availability

The datasets generated and analysed during the current study are not publicly available since the online survey was conducted by the join agreement among the Seoul Health Foundation and local governments but are available from the corresponding author on reasonable request.
